# AMPK/Ulk1-dependent autophagy as a key mTOR regulator in the context of cell pluripotency

**DOI:** 10.1038/s41419-019-1501-9

**Published:** 2019-03-18

**Authors:** Irina I. Suvorova, Valery A. Pospelov

**Affiliations:** Institute of Cytology of the Russian Academy of Sciences, St. Petersburg, Russia

The mechanisms providing high-quality control of intracellular components are critical in embryonic development and, accordingly, must be highly effective in embryonic stem cells (ESCs) generating all types of adult tissues. Recently, it has been shown that defective ESCs in embryogenesis are eliminated from the population through the mammalian target of rapamycin (mTOR)/p53 mechanism^1^. Defective ESCs are the cells that, in response to differentiation stimuli, are not able to exit from pluripotency, remaining undifferentiated. The underlying reason for the resistance to differentiation is dysregulation of signal transduction pathways leading to the mTOR pathway activation^1^. The mTOR pathway induces protein synthesis and cell growth in response to mitogenic signaling in the presence of high nutrient/energy levels and low stress. The deletion of the *mtor* gene results in embryonic lethality shortly after implantation, indicating its pivotal role in differentiation^2^. Based on this the pluripotency establishment in vivo occurs in the absence of the mTOR pathway but requires autophagy activation that is an antagonist of the mTOR activity. Autophagy is suppressed in oocytes but it is massively induced in a short time after fertilization^3^. At the same time, maternal proteins and organelles rapidly degrade and are replaced by embryonic genome-derived proteins, and consequently fertilized oocyte undergoes a large-scale intracellular rearrangement in a process called maternal-to-zygotic transition. Autophagy is responsible for this degradation, because it allows carrying out of large-scale restructuring of intracellular components by generating enough amino acids for newly synthesized proteins, in particular pluripotency-associated (PA) proteins. A key role of autophagy in the homeostasis of ESCs has already established. It was shown that autophagy regulates the levels of PA such as Oct4, Sox2, Nanog, and Klf4, and quantitative imbalances of these affect differentiation potential of cells^4^. Therefore, disturbance of autophagy may be crucial for the development of differentiation-resistant cells and their inclusion in the final ESC- or induced pluripotent stem cell (iPSC)-derived cell product that will produce tumors after transplantation. Observed loss of the mTOR activity in differentiation-resistant cells can be linked to persistent autophagy, being a barrier to exit from pluripotency.

AMP-activated protein kinase (AMPK)-dependent Ulk1 phosphorylations are a direct mechanism to induce autophagy in cells. In the last issue of *Cell Death Discovery*, Suvorova et al.^5^ identified that the AMPK/Ulk1-dependent autophagy promotes pluripotency of mouse ESCs (mESCs) under resveratrol treatment. Resveratrol is a natural polyphenolic compound showing beneficial effects on different animal models via autophagy induction. We have treated a serum-based culture of mESCs that are characterized as heterogeneous according to the degree of differentiation of individual cells by resveratrol. It was found that resveratrol makes mESC population more homogeneous and this was confirmed by morphological results and increased expressions of PA proteins Oct4, Sox2, Nanog, Klf4, and SSEA-1. Enhanced pluripotency is correlated with high autophagic flux in resveratrol-treated cells, suggesting autophagic control of pluripotency. Autophagy was executed via AMPK/Ulk1 pathway activation and mTOR complex 1 (mTORC1) pathway inhibition. Resveratrol seems to maintain pluripotency through downregulation of the mTORC1/Ulk1 pathway. In order to prove this, we had used an inhibitor of Ulk1 activity and respectively Ulk1-dependent autophagy MRT68921. According to the data obtained, resveratrol induces LC3 protein accumulation that is abrogated by Ulk1 inhibition, indicating Ulk1-dependent autophagy supression. Resveratrol-induced increment of Oct4 and Nanog expressions were also abrogated by Ulk1 inhibition. Therefore, resveratrol supports the undifferentiated state of mESCs via mTOR suppression and AMPK/Ulk1-dependent autophagy activation. AMPK activators have already shown positive effect on ESC pluripotency, suggesting the activation of Ulk1 by AMPK is enough to regulate ESC identity under normal physiological conditions^6^. We confirmed this suggestion by results obtained from the stable mESC line with doxycycline-inducible *ulk1* gene expression. Under Ulk1 activation, expressions of pluripotency factors Oct4, Sox2, Klf4, and Nanog were detected, confirming the significance of the AMPK/Ulk1 pathway activation to retain mESC pluripotency.

**Fig. 1:**
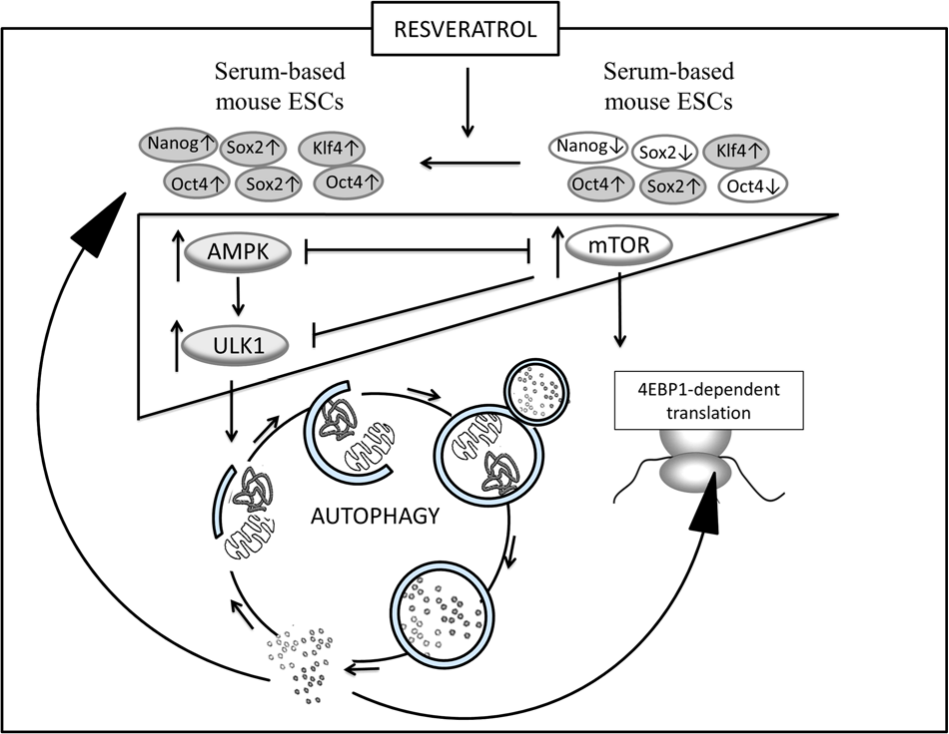
The effect of resveratrol on pluripotency of mouse embryonic stem cells (ESCs). The diagram shows how mTOR/AMPK/Ulk1 signaling axis directs mouse ESC pluripotency upon resveratrol treatment. Serum-based culture of mouse ESCs is characterized by heterogeneous expressions of Oct4, Sox2, Nanog, and Klf4 proteins and by upregulated mTOR complex 1 (mTORC1) pathway. mTORC1 directly phosphorylates and inhibits Ulk1, suppressing autophagy. Upon resveratrol treatment, abrogation of differentiation occurs by prevailing the AMPK/Ulk1 pathway activation over the mTOR pathway when the high autophagic flux maintains ESC identity by guarding their pluripotency capacity. mTOR mammalian target of rapamycin, AMPK AMP-activated protein kinase

Therefore, the AMPK/Ulk1 pathway activation and the mTOR pathway inhibition can be sensitive points to retain pluripotency and to exit from pluripotency, respectively. Recently, genome-wide CRISPR-KO (clustered regularly interspaced short palindromic repeats/knockout) screen identified a key role for FLCN (folliculin) to promote the exit from human naive pluripotency through the activation of the mTOR pathway^7^. However, FLCN is an evolutionarily conserved negative regulator of AMPK^8^. It has been shown that loss of FLCN results in constitutive activation of AMPK which induces autophagy, inhibits apoptosis, and promotes tumorogenesis^8^. For this reason FLCN knockout-naive human ESCs may remain pluripotent since they can retain high level of autophagy due to the AMPK/Ulk1 pathway upregulation. However, the AMPK/Ulk1 pathway is tightly connected with p53 activity that plays an important role in elimination of mutant cells from population. It is known that AMPK induces p53 activation in cells, suggesting apoptosis involvement if something goes wrong^6^. It can be assumed that defective embryonic stem cells in embryogenesis are marked by increased activation of p53 protein due to persistent autophagy mediated by the AMPK/Ulk1 pathway^1^. Therefore, the findings highligh a critical role for the AMPK/Ulk1-dependent autophagy to maintain ESC self-renewal and pluripotency and further perspectives to study its regulation in the context of pluripotency exit (Fig. 1).
